# Wurtzite copper-zinc-tin sulfide as a superior counter electrode material for dye-sensitized solar cells

**DOI:** 10.1186/1556-276X-8-464

**Published:** 2013-11-06

**Authors:** Jun Kong, Zheng-Ji Zhou, Mei Li, Wen-Hui Zhou, Sheng-Jie Yuan, Rong-Yue Yao, Yang Zhao, Si-Xin Wu

**Affiliations:** 1 The Key Laboratory for Special Functional Material of MOE, Henan University, Kaifeng 475004, China

**Keywords:** Counter electrode, Wurtzite, CZTS, Dye-sensitized solar cells

## Abstract

Wurtzite and kesterite Cu_2_ZnSnS_4_ (CZTS) nanocrystals were employed as counter electrode (CE) materials for dye-sensitized solar cells (DSSCs). Compared to kesterite CZTS, the wurtzite CZTS exhibited higher electrocatalytic activity for catalyzing reduction of iodide electrolyte and better conductivity. Accordingly, the DSSC with wurtzite CZTS CE generated higher power conversion efficiency (6.89%) than that of Pt (6.23%) and kesterite CZTS (4.89%) CEs.

## Background

For the advantages of low cost, environmental friendliness, easy fabrication, and light-to-energy conversion with relatively high efficiency, dye-sensitized solar cells (DSSCs) are listed as one of the most promising photovoltaic devices [1-6]. A typical DSSC has a sandwich structure: a dye-sensitized semiconductor photoanode, an electrolyte with a redox couple (triiodide/iodide), and a counter electrode (CE) catalyzing the reduction of I_3_^-^ to I^-^. The CE in photoelectrochemical solar cells plays an important role in transferring electrons from the external circuit back to the redox electrolyte for catalytic reduction of the redox electrolyte. Up to now, the most conventional CE is fluorine-doped tin oxide (FTO) glass coated with a thin layer of platinum, which has the excellent electrocatalytic activity for the reduction of charge carriers in an electrolyte as well as high conductivity. However, Pt is scarce and expensive which makes the cost of DSSCs high and limits the potential large-scale applications. To address this issue, efforts have been made to replace the Pt CE. Currently, the researches about a CE alternative were focused on two aspects. Firstly, different materials were tried to be used as CE in DSSC devices, such as carbon-based materials [7-9], conductive polymer [10,11], and inorganic semiconductor materials [12-14]. Second, for the certain given CE materials, the effect of morphology on the efficiency of DSSC devices has received much attention. For example, in carbon-based CE materials, the different morphologies, such as nanotubes [15] and mesoporous [16] and hierarchical [17] structures, were used as CE in DSSC devices. However, for a special CE material, the influence of different phases on the efficiency of DSSC has not been reported.

In our previous work [18], we prepared wurtzite and kesterite Cu_2_ZnSnS_4_ (CZTS) nanocrystals (NCs) using facile one-pot method. Hall effect measurement demonstrated that, compared to the kesterite CZTS films, the wurtzite CZTS films show a higher carrier concentration and lower resistivity. The high carrier concentration and low resistivity mean high electrical conductivity, which would result in the wurtzite CZTS which is more favorable when used as CE in DSSC. In former reports, the CZTS materials used as CEs usually possess the kesterite structure [19-21]; however, the wurtzite CZTS has not yet been reported as a CE in DSSCs.

Herein, for the first time, using CZTS NC films as CEs, we discussed the effect of wurtzite and kesterite CZTS crystal structure on the photovoltaic performance of DSSCs. Through various characterizations, such as cyclic voltammetry and electrochemical impedance spectroscopy, the obtained wurtzite CZTS NC film was demonstrated as a more effective CE material to replace the expensive Pt, yielding a low-cost, high-efficiency DSSC compared to the kesterite CZTS CE.

## Methods

### Fabrication of the CZTS thin film for CE

The synthetic process of kesterite and wurtzite CZTS NCs was similar as before [18]. The CZTS NCs were finally dissolved in tetrachloroethylene and concentrated to 10 mg/mL. Then, CZTS NC films were fabricated on a FTO glass by drop coating method using the obtained ‘nano-ink’. The thickness of the two CZTS layers prepared by dropcasting was about 2 μm. After coating, the CZTS NC films were vacuum-dried at 60°C, and then a post-annealing process was conducted in argon atmosphere at a rate of 2°C/min and held at 500°C for 30 min.

### Device assembly

Porous TiO_2_ photoanodes were immersed overnight in 0.3 mM ethanolic solution of N-719 at room temperature to absorb the dye. The TiO_2_ photoanodes were then taken out and rinsed with ethanol to remove the excess dye adsorbed and dried in air at room temperature. The sandwich-type solar cell was assembled by placing the CZTS CE on the N-719 dye-sensitized photoelectrode (working electrode) and clipped together as an open cell for measurements. The cell was then filled with a liquid electrolyte composed of 0.1 M anhydrous LiI, 0.12 M I_2_, 1.0 M 1,2-dimethyl-3-*n*-propylimidazolium iodide (DMPII), and 0.5 M *tert*-butylpyridine in dehydrated acetonitrile by capillary force.

## Results and discussion

**Figure 1:**
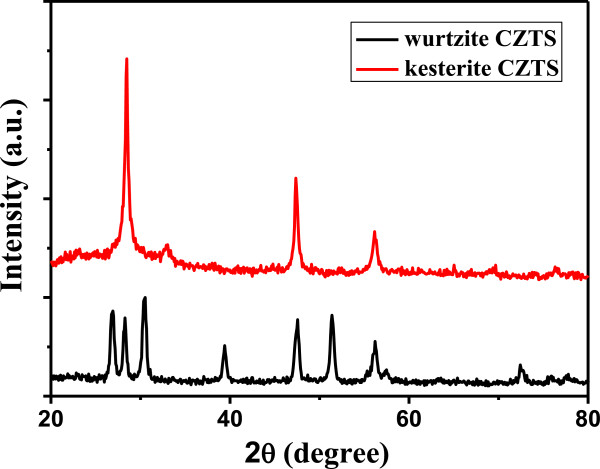
X-ray diffraction patterns of the as-obtained CZTS thin films after annealing.

Crystal structures of the CZTS thin films after annealing were confirmed by XRD patterns (Figure 1). The major diffraction peaks of the kesterite CZTS thin film can be indexed to kesterite CZTS (JCPDS 26–0575) [22-24] (red curve) and to cation-disordered wurtzite CZTS [25] (black curve), respectively. No characteristic peaks of other impurities are detected, such as ZnS, CuS, or Cu_2_S.

**Figure 2:**
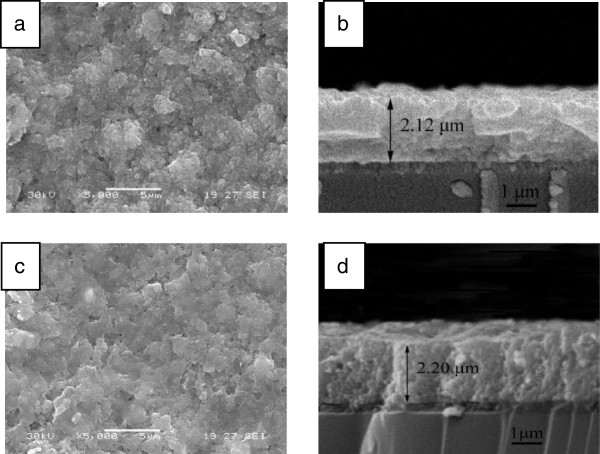
**SEM images of CZTS NCs films. (a)** Top view and **(b)** cross section of the wurtzite film. **(c)** Top view and **(d)** cross section of the kesterite film with sintering at 500°C for 30 min.

Figure 2 shows scanning electron microscopy (SEM) images of the cross section of the kesterite (d) and wurtzite (b) CZTS thin films with sintering at 500°C for 30 min, respectively. Since these CZTS films were composed of CZTS NCs, the films possess a relatively high surface area (Additional file 1: Figure S1). From the images of the cross section, we can observe that the CZTS films were very dense and compact without cracks. The thickness of two CZTS films was about 2 μm. The SEM results illuminated that the thickness and compactness of the wurtzite and kesterite CZTS films were very similar in our experiments.

**Figure 3:**
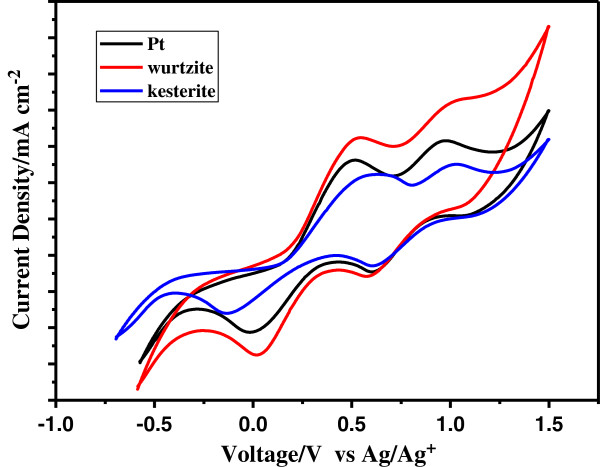
**Cyclic voltammograms of different CEs with a scan rate of 50 mV s**^**-1**^**.**

The electrocatalytic activity of CZTS CEs under the I^-^/I_3_^-^ electrochemical system using a three-electrode system was investigated by cyclic voltammetry (CV) (shown in Figure 3). The cyclic voltammograms of I^-^/I_3_^-^ redox reaction on different CZTS CEs are similar; two pairs of redox peaks (Ox-1/Red-1, Ox-2/Red-2) are observed. As we knew, the peak currents and the peak-to-peak (Ox-1 to Red-1) separation (Epp) are two important parameters for catalytic activities [26-28]. From Figure 3 and Table 1, the higher peak current density and lower Epp value reveal that the wurtzite CZTS film as CE material is a remarkable electrochemical catalyst for the reduction of I_3_^-^, even better than the Pt CE. At the same time, the lower peak currents and larger Epp illustrate that the electrocatalytic activity of the kesterite CZTS is inferior to that of wurtzite CZTS. Since all of the Epp are more than 30 mV, the reaction of the I^-^/I_3_^-^ redox couple at the CE/electrolyte interface should be a quasi-reversible electrode process.

**Table 1:** Photovoltaic parameters and fitted impedance parameters

**CEs**	**Thickness (μm)**	***J***_**sc**_**(mA/cm**^**2**^**)**	***V***_**oc**_**(V)**	**FF (%)**	**PCE (%)**	***R***_**s**_**(Ω cm**^**2**^**)**	***R***_**ct**_**(Ω cm**^**2**^**)**	**Epp (V)**
Pt	0.10	11.43	0.78	69.84	6.23	15.91	2.92	0.536
Wurtzite	2.12	13.41	0.75	68.69	6.89	16.20	2.78	0.528
Kesterite	2.20	10.20	0.73	65.72	4.89	17.02	3.56	0.760

Photovoltaic parameters for various DSSCs fabricated using different counter electrodes and the fitted impedance parameters extracted from fabricated symmetric cells are as follows: *J*_sc_, short-circuit current density; *V*_*oc*_, open-circuit voltage; *FF*, fill factor; *R*_*s*_, series resistance; *R*_*ct*_, charge transfer resistance.

**Figure 4:**
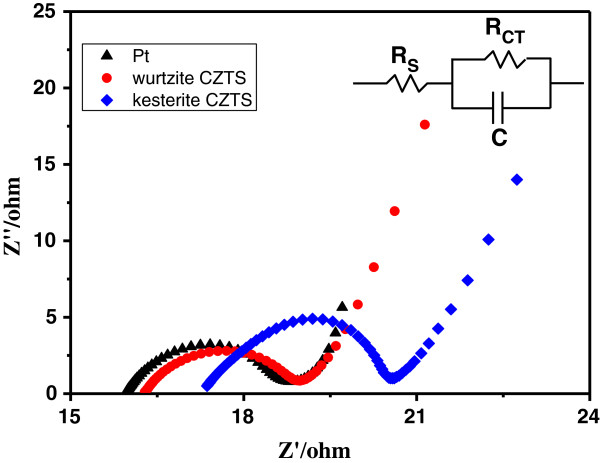
**Nyquist plots for different CEs.** The test was performed with the symmetrical cells fabricated with two identical electrodes.

**Figure 5:**
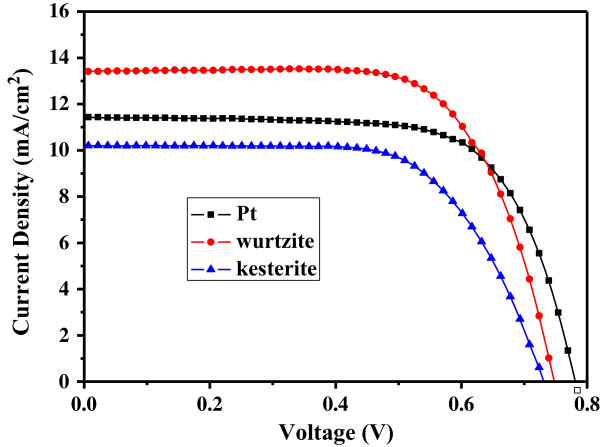
**Current density-voltage (*J*-*V*) curves of DSSCs based on different CEs under AM 1.5 (100 mW cm**^**-2**^**).**

The performance of CE materials in DSSC devices depends not only on its catalytic activity, but also on the electrical conductivity [29,30]. Electrochemical impedance spectroscopy (EIS) is an effective and widely used tool for investigating the charge transfer process and thereby for evaluating the catalytic activity of a catalyst [31]. Figure 4 shows the Nyquist plots for the devices with wurtzite and kesterite CZTS CEs. The high-frequency intercept on the real axis corresponds to the series resistance (*R*_s_). The first semicircle at the high-frequency region arises from the charge transfer property (*R*_ct_). The values of *R*_s_ and *R*_ct_ obtained by fitting the spectra in Figure 5 with an EIS spectrum analyzer are summarized in Table 1. The largest *R*_s_ (17.02 **Ω**) of kesterite CZTS CE can be attributed to the strong ligand of oleylamine on the CZTS NC surface. Similarly, some organic substance capped on the surface of the wurtzite CZTS NCs made the *R*_s_ (16.2 **Ω**) of wurtzite CZTS CE higher than that (15.91 **Ω**) of Pt CE. However, the value of *R*_ct_ (2.78 **Ω**) of the wurtzite CZTS CE is lower than that of Pt (2.92 **Ω**) and kesterite CZTS (3.56 **Ω**). The smallest *R*_ct_ for wurtzite CZTS CE implies that it has eximious catalytic activity on the reduction of triiodide and supersedes the expensive Pt as the CE in DSSCs. The conclusions for the catalytic activity derived from the EIS and CV data are consistent.

Figure 5 shows the photocurrent density-voltage (*J*-V) curves of these DSSCs with different CE materials, and the detailed photovoltaic parameters are summarized in Table 1. For the DSSC using the kesterite CZTS CE material, the power conversion efficiency (*η*) of the device was relatively low (4.89%), since the data of photovoltaic parameters such as*J*_sc_, *V*_oc_, and FF were low (*J*_sc_ = 10.20 mA/cm^2^, *V*_oc_ = 0.73 V, FF = 65.72%, respectively). For the wurtzite CZTS CE material, the efficiency of the DSSC device was high (6.89%); the high performance resulted from the improved photovoltaic parameters, such as*J*_sc_, *V*_oc_, and FF (*J*_sc_ = 13.41 mA/cm^2^, *V*_oc_ = 0.75 V, FF = 68.69%, respectively). The efficiency of the DSSC using wurtzite CZTS CE was even better than that of Pt CE (*η* = 6.23%, *J*_sc_ = 11.43 mA/cm^2^). The values of *V*_oc_were almost constant in these DSSC devices using different CE materials. The difference of the efficiency of DSSC devices mainly resulted from the parameters of *J*_sc_ and FF. The high FF of the wurtzite CZTS CE may be attributed to its relatively low *R*_s_[32]. The highest *J*_sc_ for wurtzite CZTS should come from its high carrier concentration and low resistivity. According to our previous result, the Hall effect measurement demonstrated that compared to the kesterite CZTS films, the wurtzite CZTS films show a higher carrier concentration and lower resistivity [18]. Wurtzite CZTS is a hexagonal crystal system and metastable; perhaps, this structure is beneficial for catalysis and charge conductivity. The *J*-*V* results signify that the wurtzite CZTS could be a somewhat economical and effective CE material for DSSC.

## Conclusions

In this work, we used the wurtzite and kesterite CZTS NC films as effective CEs in DSSCs. The measurement of the photovoltaic performance of DSSCs showed that the wurtzite CZTS CE exhibited higher solar energy conversion efficiency (6.89%). The results of CV and EIS demonstrated the superior electrocatalytic activity of the wurtzite CZTS NC films. The excellent performance of the wurtzite CZTS CE paves a new pathway for preparing cheap and highly efficient CEs for DSSCs.

## Competing interests

The authors declare that they have no competing interests.

## Authors’ contributions

JK carried out the experiments, characterization, and acquisition of data. ZJZ participated in the designing of the experiments, experiment analysis, interpretation of data, and language modification. ML and WHZ carried out the sample preparation and measurements. SJY, RYY, and YZ participated in the discussion. SXW is the investigator who helped in the analysis and interpretation of data, drafting of the manuscript, and revisions. All authors read and approved the final manuscript.

## Supplementary Material

Additional file 1: Figure S1 N_2_ adsorption-desorption isotherms of wurtzite CZTS NCs and kesterite CZTS NCs at 77 K. Click here for file
