# Supplementary figures and images for: Wurtzite copper-zinc-tin sulfide as a superior counter electrode material for dye-sensitized solar cells
Source: Nanoscale Res Lett. 2013 Nov 6;8(1):464. doi: 10.1186/1556-276X-8-464 (PMC4228334; doi:10.1186/1556-276X-8-464)

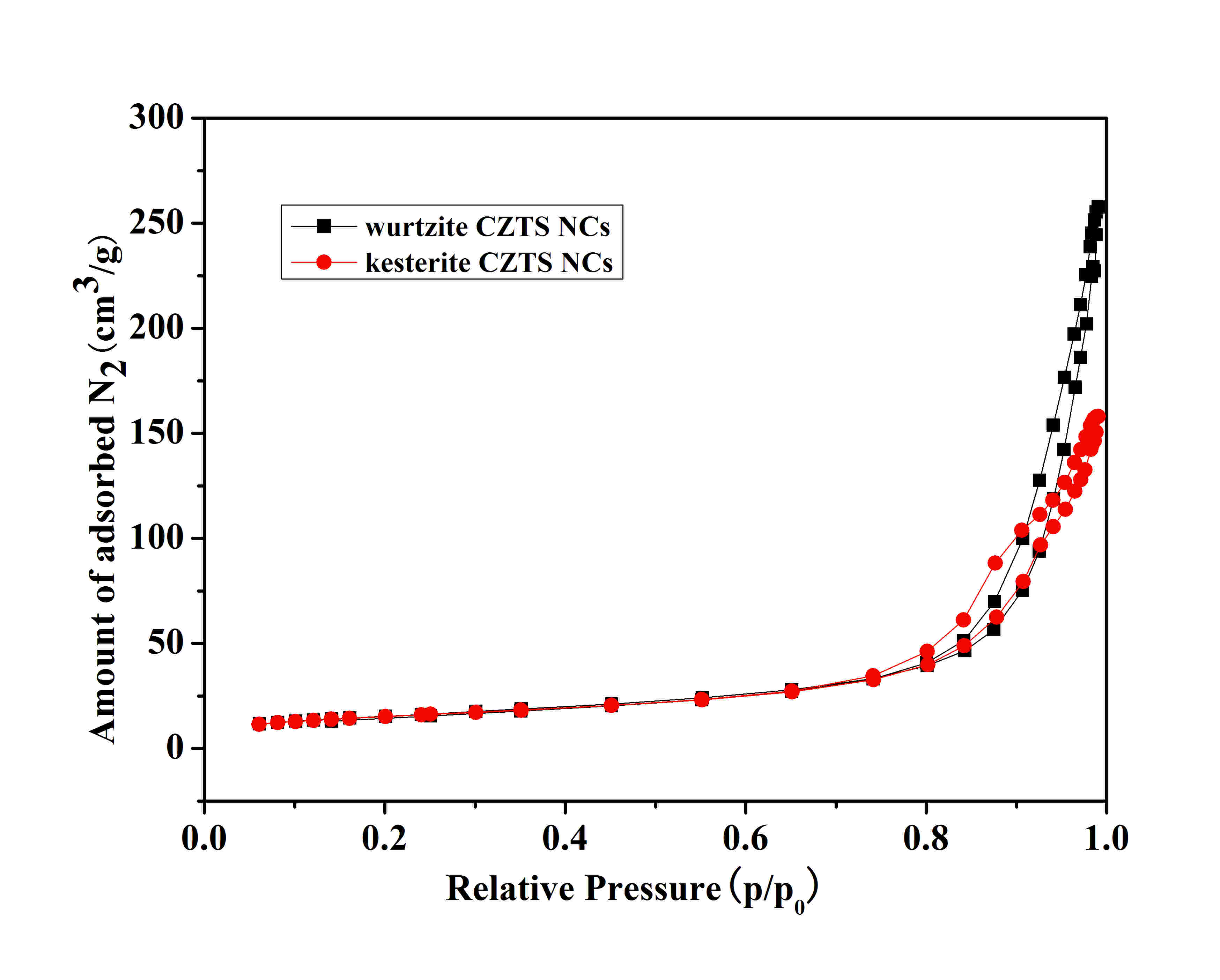
**Figure S1.** N2 adsorption-desorption isotherms of wurtzite CZTS NCs and kesterite CZTS NCs at 77 K.

Supplement: Additional file 1: Figure S1 — N2 adsorption-desorption isotherms of wurtzite CZTS NCs and kesterite CZTS NCs at 77 K. [file 1556-276X-8-464-S1.doc]
